# The neuromyelitis optica presentation and the aquaporin-4 antibody in HIV-seropositive and seronegative patients in KwaZulu-Natal, South Africa

**DOI:** 10.4102/sajhivmed.v18i1.684

**Published:** 2017-01-31

**Authors:** Ahmed I. Bhigjee, Anandan A. Moodley, Izanne Roos, Cait-Lynn Wells, Pratistadevi Ramdial, Monika Esser

**Affiliations:** 1 Department of Neurology, Nelson R Mandela School of Medicine, University of KwaZulu-Natal, South Africa; 2 Department of Neurology, Greys Hospital, Pietermaritzburg, South Africa; 3 Department of Neurology, University of KwaZulu-Natal, South Africa; 4 Department of Neurology, Inkosi Albert Luthuli Central Hospital, University of KwaZulu-Natal, South Africa; 5 Department of Neurology, Nelson R. Mandela School of Medicine, University of KwaZulu-Natal, South Africa; 6 Department of Anatomical Pathology, NHLS, Inkosi Albert Luthuli Central Hospital, South Africa; 7 Immunology Unit, NHLS Tygerberg, Tygerberg Hospital, South Africa

## Abstract

**Background:**

The association of the anti-aquaporin-4 (AQP-4) water channel antibody with neuromyelitis optica (NMO) syndrome has been described from various parts of the world. There has been no large study describing this association from southern Africa, an HIV endemic area. HIV patients often present with visual disturbance or features of a myelopathy but seldom both either simultaneously or consecutively. We report our experience of NMO in the era of AQP-4 testing in HIV-positive and HIV-negative patients seen in KwaZulu-Natal, South Africa.

**Methods:**

A retrospective chart review was undertaken of NMO cases seen from January 2005 to April 2016 in two neurology units serving a population of 7.1 million adults. The clinical, radiological and relevant laboratory data were extracted from the files and analysed.

**Results:**

There were 12 HIV-positive patients (mean age 33 years), 9 (75%) were women and all 12 were black patients. Of the 17 HIV-negative patients (mean age 32 years), 15 (88%) were women and 10 (59%) were black people. The clinical features in the two groups ranged from isolated optic neuritis, isolated longitudinally extensive myelitis or combinations. Recurrent attacks were noted in six HIV-positive patients and six HIV-negative patients. The AQP-4 antibody was positive in 4/10 (40%) HIV-positive patients and 11/13 (85%) HIV-negative patients. The radiological changes ranged from longitudinal hyperintense spinal cord lesions and long segment enhancing lesions of the optic nerves. Three patients, all HIV-positive, had tumefactive lesions with incomplete ring enhancement.

**Conclusion:**

This study confirms the presence of AQP-4-positive NMO in southern Africa in both HIV-positive and HIV-negative patients. The simultaneous or consecutive occurrence of optic neuritis and myelitis in an HIV-positive patient should alert the clinician to test for the AQP-4 antibody. It is important to recognise this clinical syndrome as specific therapy is available. We further postulate that HIV itself may act as a trigger for an autoimmune process.

## Introduction

Neuromyelitis optica (NMO) or Devic’s disease is a central nervous system inflammatory disorder originally described as a monophasic illness but recurrent cases have been noted.^1^ NMO has been described in different ethnic and racial groups worldwide.^2^

One of earliest reported cases of NMO in South Africa was by Hift and Moodley.^3^ Cosnett speculated that NMO might be a variant of multiple sclerosis (MS) in black people.^4^ Dean et al. in describing MS in black South Africans and Zimbabweans clearly included cases of NMO in their case series.^5^ A link between NMO and pulmonary tuberculosis was suggested in 1990 and more recently in 2014.^6,7^ Modi et al. recognised the opticospinal presentation as a distinct disorder in black patients.^8^ A recent global study reported that the prevalence of NMO in South Africa is 5/100 000, which together with Paraguay is the highest in the world.^9^ In 1991, Wingerchuk et al. studied the clinical course of NMO of 71 patients and devised a set of criteria for a more accurate diagnosis of NMO.^10^

HIV-positive patients commonly present with visual disturbance or myelopathy, but seldom both either simultaneously or consecutively. When we see this combination, we consider the possibility of NMO. We, therefore, undertook a retrospective analysis of all NMO cases seen in the neurology units based at Inkosi Albert Luthuli Central Hospital in Durban and Greys Hospital in Pietermaritzburg from January 2005 to April 2016. The two units serve an adult population (over the age of 15 years) of approximately 7.1 million in KwaZulu-Natal.^11^

## Patients and methods

The records of patients with the diagnosis of NMO were reviewed from each hospital’s repository. For each patient the clinical, laboratory and radiological data were extracted and entered into an Excel spreadsheet. Although a number of laboratory tests were performed on each patient, the focus was on the following tests: HIV and human T-lymphotropic virus 1 (HTLV-1) antibodies, rapid plasma reagin (RPR), anti-nuclear factor (ANF), serum AQP-4 antibody, serum angiotensin converting enzyme (SACE), routine CSF chemistry and cell count, CSF microscopy, culture and PCR for tuberculosis, CSF fluorescent treponemal assay (FTA) and oligoclonal bands in the CSF.

In the HIV-positive patients, the CSF was tested for cryptococcal antigen, together with PCR for varicella zoster (VZV), herpes simplex (HSV), cytomegalovirus (CMV) and enteroviruses. The CD4 count and viral load were also included where available.

The MRI scans were reviewed for spinal and brain lesions by Ahmed I. Bhigjee and Anandan A. Moodley. The AQP-4 antibody test was performed by a private laboratory in three cases but the rest were performed at a National Health Laboratory in the Western Cape. The cell-based Euroimmun AG (Lübeck, Germany) assay was used at both centres. This test has a claimed sensitivity and specificity of 80% and 100%, respectively (Euroimmun AG package insert).

## Ethical consideration

The study was approved by the Biomedical Research Ethics Committee of the University of KwaZulu-Natal.

## Results

A total of 12 HIV-positive patients and 17 HIV-negative patients were identified. Their demographic details are summarised in Table 1.

**TABLE 1:** Demographic data.

Variable	Total group†		HIV-positive‡		HIV-negative§	
	Number	%	Number	%	Number	%
**Age**						
Mean (SD) years	33 ± 9.7	-	33 ± 9.1	-	33 ± 10.3	-
**Gender**						
Female	24	83	9	75	15	88
**Race**						
Black	22	76	12	100	10	59
Indian	5	17	-	-	5	29
Mixed race	2	7	-	-	2	12

† *n* = 29;

‡ *n* = 12;

§ *n* = 17.

In the HIV-positive group, 9/12 (75%) were women. The age range varied from 19 to 42 years (mean ± SD 33 + 9.7). All patients were black people. The duration of illness at the time of first presentation varied from 2 weeks to 2 years in 11 patients and not stated in one. The first presentation was visual impairment in six, spinal in five and not stated in one. Recurrent attacks were noted in six patients.

In the HIV-negative group, 15/17 (88%) were women. The age range varied from 17 to 58 years (mean ± SD 33 ± 10.3). There were ten black patients, five Indians and two patients of mixed ancestry. The duration of illness at the time of first presentation varied from 5 days to 14 years. The first presentation was visual impairment in six and spinal in the rest. Recurrent attacks were recorded in six patients.

Routine laboratory tests were unhelpful in both groups of patients except for other autoantibodies. The more relevant laboratory results are summarised in Table 2. In the HIV-positive group, one patient has a positive ANF at a titre of 1:2560 with positive anti-dsDNA. In the HIV-negative group, four had a positive ANF with titres ranging from 1:50 to 1:640. Two had a positive anti-Ro antibody as well. There was a third patient who was ANF-negative but anti-Ro positive. One patient had anti-GAD antibodies and another patient was anti-Ma2 antibody positive. None of the ANF-positive patients exhibited any clinical features of systemic lupus erythematosus (SLE).

In the HIV-positive group, the anti-AQP-4 antibody test was performed in ten, four of whom returned a positive result. HTLV-1 antibodies were not detected in any of the 10 patients tested. Oligoclonal bands were absent in all six patients who were tested.

**TABLE 2:** Laboratory data.

Variable	Total†		HIV-positive‡		HIV-negative§	
	Parameter	%	Parameter	%	Parameter	%
**CSF**						
PMN/µL	0.4 ± 1.3*	-	0.16 ± 0.6*	-	0.5 ± 1.6*	-
Lymphocytes/µL	4.5 ± 9.5*	-	7 ± 12.6*	-	3.3 ± 6.5*	-
RBC/µL	39 ± 190*	-	4.2 ± 14.4*	-	65 ± 250*	-
Protein g/L	0.5 ± 0.3*	-	0.5 ± 0.3*	-	0.5 ± 0.3*	-
Glucose mmol/L	3.3 ± 0.8*	-	3.2 ± 0.3*	-	3.5 ± 1.1*	-
**OCB**						
Negative	16	55	-	-	-	-
Not performed	13	45	-	-	-	-
**GeneXpert**						
Negative	17	59	-	-	-	-
Not performed	12	41	-	-	-	-
Culture negative	29	100	-	-	-	-
CLAT negative	29	100	-	-	-	-
**FTA**						
Negative	25	86	12	100	13	76
Positive (VDRL negative)	1	3	0	-	1	6
Not done	3	10	0	-	3	18
**Serum aquaporin-4 antibody**					
Positive	15/23	65	4/10	40	11/13	85
Not performed	6/29	21	2/12	17	4/17	24
**Other autoantibodies**						
None	19	-	8	-	11	-
ANF-positive	5	-	1	-	4	-
Anti-Ro positive	3	-	0	-	3	-
Anti-GAD	1	-	0	-	1	-
Anti-Ma2/Ta	1	-	0	-	1	-
**HTLV-1**						
Positive	1/24	4	0	-	1/14	7
Not performed	5/29	17	2/12	17	3/17	18

* Mean ± SD.

† *n* = 29;

‡ *n* = 12;

§ *n* = 17.

ANF, anti-nuclear factor; CLAT, cryptococcal latex agglutination test; FTA, fluorescent treponemal assay; HTLV, human T-lymphotropic virus; OCB, oligoclonal bands.

In the HIV-negative group, of the 13 patients in whom the test was performed, 11 (85%) had anti-AQP-4 antibodies. Six of these eleven AQP-4 antibody positive cases had recurrent attacks. Three of the patients in whom the test was not performed were seen at a time when the test was not available to us. One of these patients, a 53-year-old black woman, was HTLV-1 positive. Oligoclonal bands were absent in all 10 patients who had the results of this test available.

The neuro-imaging findings are summarised in Table 3.

**TABLE 3:** Neuro-imaging findings.

Imaging	HIV-positive†		HIV-negative‡	
	Number	%	Number	%
**MRI brain**				
Not performed	2	-	3	-
Normal	3/10	30	4/14	29
Non-specific WM hyperintensity	2/10	20	1/14	7
Periventricular hyperintensity	2/10	20	4/14	29
Medullary hyperintensity	-	-	6/14	43
Tumefactive incomplete rim enhancing lesions	3/10	30		
**MRI optic nerves**				
Not done	2	-	3	-
Normal	8/10	80	7/14	50
Thickening	1/10	10	3/14	21
Enhancement	1/10	10	5/14	36
**MRI spine**				
Not done	2	-	0	-
Normal	0	-	2/17	12
Longitudinal extensive myelitis	8/10	80	13/17	76
Atrophy	1/10	10	2/17	12
Patchy multilevel hyperintensities	1/10	10	0	-

Note: As some patients had more than one change, the numbers do not all tally.

† *n* = 12;

‡ *n* = 17.

**FIGURE 1:**
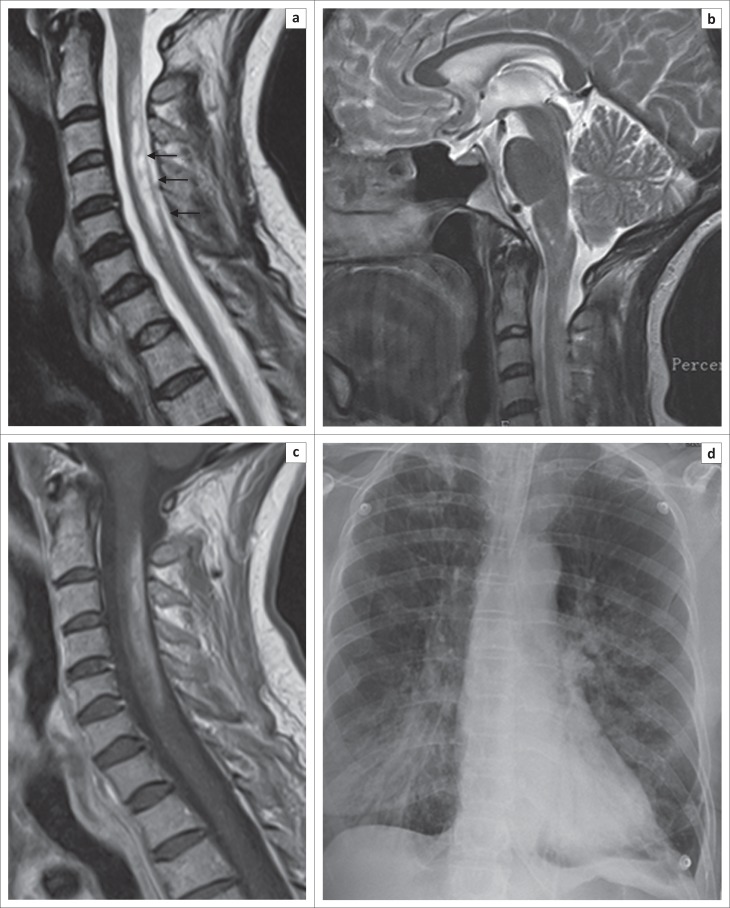
(a) and (b) are T2 sagittal images showing high intensity signal in the lower medulla and upper cervical cord; (c) is post-contrast T1 image showing enhancement of the lesion; (d) is a chest radiograph showing features consistent with tuberculosis.

**FIGURE 2:**
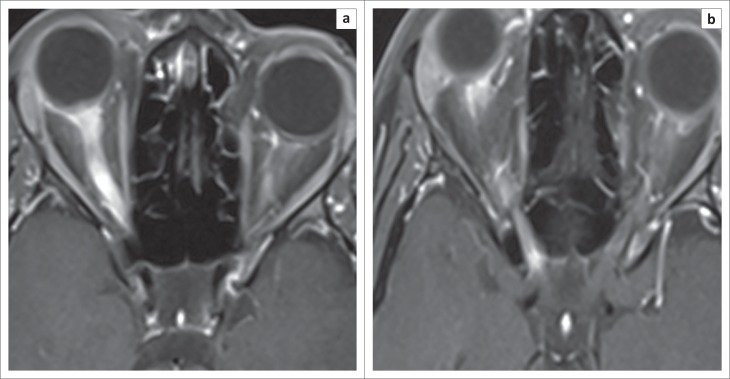
a) and (b) are orbital MRI views post-contrast. They show diffuse enhancement of the right optic nerve extending to the chiasm (arrows). Minimal enhancement of the left optic nerve is also present (right arrow in (b)).

The MRI features ranged from typical caudal medulla to upper cervical hyperintensity (Figure 1a–c), thoracic longitudinally extensive transverse myelopathy (LETM), cord atrophy and long segment enhancement of the optic nerve. For example, Figure 2a and b shows intense enhancement of the right optic nerve extending to the chiasm. There was minimal enhancement of the left optic nerve.

**FIGURE 3:**
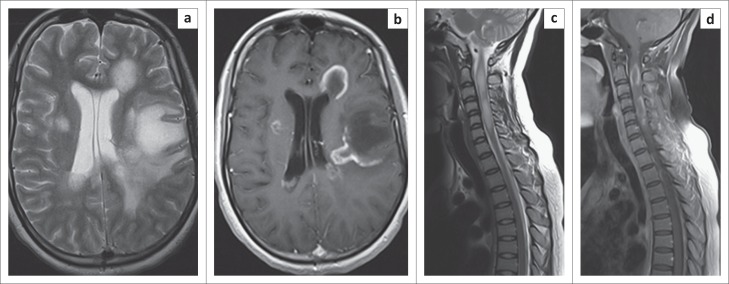
Images from an HIV-positive patient (a) and (b) are T2 and T1 post-contrast images of the brain, respectively, showing tumefactive lesions. Figure (c) and (d) are corresponding images of the spinal cord.

In the HIV-positive group, three of the brain MRI scans showed tumefactive lesions (e.g. Figure 3a and b). In none of these patients was there fever or specific cerebral symptoms such as confusion and seizures to suggest a diagnosis of acute disseminated encephalomyelitis.

## Illustrative cases

### Case 1 (HIV-negative)

A 55-year-old woman of mixed ancestry presented in March 2011 with a 3-month history of acute onset of nausea, vomiting, diplopia and incoordination of her limbs. Examination revealed normal visual acuity and optic fundi. The tone, power and tendon reflexes were normal. Joint position sense was impaired at the toes, vibration sense was abnormal up to the anterior superior iliac spine and there was patchy pinprick sensory loss in the legs. There was bilateral horizontal gaze evoked nystagmus and incoordination of all four limbs.

The MRI showed an irregular hyperintense lesion from the medulla to the C3 vertebral level (Figure 1a). There was minimal enhancement of the lesion. The CSF was acellular with a protein of 0.54 g/L and a glucose level of 3.3 mmol/L. Oligoclonal bands were not detected in the CSF. Whilst in the ward, the patient spontaneously started to improve. No treatment was instituted. When reviewed 4 months later, she was much better and ambulant with mild disability, and a repeat MRI showed shrinkage of the medullary cervical lesion.

She presented again in April 2014 with acute worsening of her incoordination and loss of sensation in the right hand. Examination revealed marked bilateral incoordination, normal tone and power but depressed tendon jerks in the right arm and knee. A repeat MRI showed worsening of the cervico-medullary lesion with extension now down to the C6 vertebral level (Figure 1b). Enhancement was present (Figure 1c). A repeat CSF was again not helpful. Serum ACE was normal. HIV test was negative. The ANF was positive 1:640 with a speckled pattern. The double-stranded anti-DNA and anti-ENA were negative. The AQP-4 antibody was negative. A chest radiograph showed cavitatory lesion in both lung fields. (Figure 1d). The GeneXpert test on a bronchial aspirate was positive for *Mycobacterium tuberculosis*. The patient was started on antituberculous therapy and given a 5-day course of 500 mg intravenous methylprednisolone followed by oral prednisone. The patient improved. At follow-up, she maintained her improvement.

In December 2014, she was admitted quadriplegic, pyrexial and in respiratory distress. *Klebsiella pneumoniae* was isolated from a bronchial aspirate. The AQP-4 antibody was now positive. The patient was intubated, ventilated and given appropriate antibiotics but died soon thereafter.

### Case 2 (HIV-positive)

A 40-year-old HIV-seropositive woman on antiretroviral therapy presented in 2016 with weakness of the arms, shock-like pain in the limbs and cramps in the legs for about 3 months. In 2014, she had an episode of left-sided weakness which partially resolved. She had no visual symptoms. On examination, her mental state and cranial nerves were normal. The visual acuity in both eyes was normal. Power in the right arm and leg was normal. On the left side, there was a 4/5 weakness in a pyramidal distribution. The tendon reflexes were brisker on the left. The abdominal reflexes were absent and both plantar responses were equivocal. The outstretched arms demonstrated ‘piano playing’ with gross proprioceptive loss. There was patchy pinprick impairment in the limbs. There were no features to suggest SLE. Routine blood tests were normal apart from mildly deranged liver enzymes and an ANF titre of 1:2560. The CD4 count was 712 cells/µL. The AQP-4 antibody was positive. The MRI of the spine showed a hyperintense signal extending from the caudal medulla to C7. In the axial slices, the hyperintensity was posteriorly located. The MRI of the brain was normal. The CSF was acellular, with a glucose level of 3.5 mmol/L and protein level of 0.36 g/L. The PCR for viruses was negative. Other tests that were negative in the CSF included smear, GeneXpert and culture for tuberculosis, cryptococcal antigen and the CSF FTA. The patient was commenced on prednisone and azathioprine and was due for a follow-up appointment.

## Discussion

### HIV-seropositive group

There were 12 HIV-seropositive patients who had a NMO presentation. The anti-AQP-4 antibody was positive in 4 of 10 patients tested. There have been isolated reports of HIV and NMO presentations, summarised in Table 4.

**TABLE 4:** Literature survey of neuromyelitic presentation in HIV-seropositive patients.

Reference	No. of patients	Country of origin	Anti-AQP-4 antibody test
Blanche et al.^12^	1	Democratic Republic of Congo	ND
Modi et al.^13^	2	South Africa	ND
Salazar et al.^14^	1	USA (Caucasian)	Positive
Feyissa et al.^15^	2	USA (both African American)	1 of 2 tested was positive
Our study	12	South Africa	4 of 10 tested were positive

ND, not done.

Although visual disturbance in HIV infection is common, the underlying cause is usually an opportunistic infection. Examples include CMV, HSV, VZV, toxoplasmosis, syphilis, cryptococcal and candida infection. In all these cases, the underlying problem is a neuroretinitis or uveoretinitis. Isolated optic neuritis or optic neuropathy is rare.^16,17,18^ Some have been shown to respond to steroids or highly active anti-retroviral therapy (HAART).^16,17,18^ Yet others may develop optic neuropathy whilst on HAART especially stavudine and didanosine. In these instances, it is believed that HAART unmasks Leber’s hereditary optic neuropathy.^19^ In contrast, the optic neuritis in the NMO cases is usually severe, does not respond to HAART or may partially respond to steroids.

Similarly, the cause of an LETM in an HIV-seropositive patient is usually an opportunistic infection. Examples include herpes virus infection, tuberculosis, syphilis and HTLV-1.^20^ HIV itself may cause a transverse (short segment) partial myelopathy at seroconversion and the well-documented vacuolar myelopathy. LETM, if it does occur, must be a very rare manifestation of HIV itself.

It follows then that the co-occurrence of optic neuritis and LTEM because of HIV itself is unlikely. There are several possible explanations for the NMO presentation in HIV-positive patients. Because of high HIV prevalence in the population, the patients may have anti-AQP-4-related NMO and incidental HIV infection. The four AQP-4-positive patients we describe above may fall into this category. Yet others may have the same autoimmune-related NMO but maybe seronegative for AQP-4 antibody, as the test has a variable sensitivity of 70% – 80%.^21,22^ Some of the patients may have the uncommon anti-myelin oligodendrocyte glycoprotein (MOG)-mediated disease which presents in a similar manner. We did not test for this antibody.

Another postulate is that HIV acts as a trigger for the autoimmune process. This has been seen with pulmonary tuberculosis^6,7^ and antecedent or recent viral infections.^10,23,24^

### HIV-seronegative group

All 17 patients fulfilled the NMO diagnostic criteria for adult patients^25^ and none had the imaging ‘red flags’ suggestive of MS.^25^ Further, MS is rare in black subjects. The clinical course in this group of patients was typical and recurrent attacks were noted in 6 of the 17 patients. Anti-AQP-4 antibodies were detected in 11 of 13 patients tested. Four of these patients were noted to have recurrent attacks, and six had a monophasic illness at the time of the last follow-up. The two anti-AQP-4 antibody-negative patients were a man who developed a testicular tumour and a woman who had recurrent attacks. Anti-MOG antibody testing was not available.^26^

A 53-year-old woman who did not have an anti-AQP-4 antibody test was seropositive for HTLV-1. This must be a very rare presentation as we could find only one report, and in our experience of over 200 cases of HTLV-1-associated myelopathy, we have not had a single patient with an opticospinal presentation.^27^

### NMO as a paraneoplastic presentation

Following the discovery of the anti-AQP-4 antibody, Pittock et al. identified a thymic carcinoma in an NMO patient.^28^ A subsequent review by the Mayo Group found an association between NMO and a variety of other malignancies including breast, bronchus, thyroid, carcinoid and lymphoma.^29^ The Indian man in our series presented with NMO first and became aware of a testicular mass about 17 months later. The anti-AQP-4 antibody test performed at the time of his neurological presentation was negative. The neurological deficits improved on steroids and azathioprine. He underwent orchidectomy and chemotherapy and currently has no evidence of tumour. His anti-Ma2 antibody test was positive. An underlying malignancy should be considered in any patient with an unexplained NMO presentation.

## Conclusion

Our study has all the limitations of a retrospective analysis, the most important being that of missing data. It does not have long-term follow-up on some of the patients. Thus, we cannot be certain whether each had a monophasic or recurrent illness. We did not test for anti-MOG antibodies which occurs in some NMO patients who are negative for anti-AQP-4 antibodies.^26^

Nonetheless, this study is probably the largest one to emerge from Africa in the HIV era where anti-AQP-4 testing is available. Recognition of the NMO presentation is important as patients respond to immunosuppressant and immunomodulatory therapy. Ongoing therapy is also necessary to prevent recurrent attacks.

## References

[1] Jarius S, Wildemann B The history of neuromyelitis optica. J Neuroinflammation. 2013;10:8 http://dx.doi.org/10.1186/1742-2094-10-8 23320783 10.1186/1742-2094-10-8 PMC3599417

[2] Pandit L, Asgari N, Apiwattanakul M, et al Demographic and clinical features of neuromyelitis optica: A review. Mult Scler J. 2015;21(7):845–853. http://dx.doi.org/10.1177/1352458515572406 10.1177/1352458515572406 PMC4463026 25921037

[3] Hift W, Moodley T A possible case of neuromyelitis optica in a Bantu patient. S Afr Med J. 1973;47(23):987–988. 4714284

[4] Cosnett JE Multiple sclerosis and neuromyelitis optica. Case report and speculation. S Afr Med J. 1981;60(6):249–51. 7256475

[5] Dean G, Bhigjee AI, Bill PLA, et al Multiple sclerosis in black South Africans and Zimbabweans. J Neurol Neurosurg Psychiatry. 1994;57:1064–1069. http://dx.doi.org/10.1136/jnnp.57.9.1064 8089669 10.1136/jnnp.57.9.1064 PMC1073127

[6] Silber MI, Willcox PA, Bowen RM, Unger A Neuromyelitis optica (Devic’s syndrome) and pulmonary tuberculosis. Neurology. 1990;40(6):934–938. http://dx.doi.org/10.1212/WNL.40.6.934 2345617 10.1212/wnl.40.6.934

[7] Zatjirua V, Butler J, Carr J, Henning F Neuromyelitis optica and pulmonary tuberculosis: A case control study. Int J Tuberc Lung Dis. 2011;15(12):1675–1680. http://dx.doi.org/10.5588/ijtld.10.0780 22118177 10.5588/ijtld.10.0780

[8] Modi G, Mochan A, Modi M, Saffer D Demyelinating disorder of the central nervous system occurring in black South Africans. J Neurol Neurosurg Psychiatry. 2001;70:500–505. http://dx.doi.org/10.1136/jnnp.70.4.500 11254774 10.1136/jnnp.70.4.500 PMC1737298

[9] World wide prevalence of NMO [homepage on the Internet]. c2015 [cited Dec 7]. Available from http://www.msif.org

[10] Wingerchuk DM, Hogancamp WF, O’Brien PC, Weinshenker BG The clinical course of neuromyelitis optica (Devic’s syndrome). Neurology. 1999;53:1107–1114. http://dx.doi.org/10.1212/WNL.53.5.1107 10496275 10.1212/wnl.53.5.1107

[11] Mid year population estimates Statistical release P0302. 2015 [cited 2015 Dec 7]. Available from: www.statssa.gov.za

[12] Blanche P, Diaz E, Gombert B, Sicard D, Rivoal O, Brezin A Devic’s neuromyelitis optica and HIV-1 infection. J Neurol Neurosurg Psychiatry. 2000;68:795–796. http://dx.doi.org/10.1136/jnnp.68.6.795a 10.1136/jnnp.68.6.795a PMC1736953 10877625

[13] Modi G, Ranchod J, Hari K, Mochan A, Modi M Non-traumatic myelopathy at the Chris Hani Baragwanath Hospital, South Africa – The influence of HIV. QJM. 2011;104:697–703. http://dx.doi.org/10.1093/qjmed/hcr038 21441578 10.1093/qjmed/hcr038

[14] Salazar R, Cerghet M, Shad A, Markowitz NP NMO-IgG positive relapsing longitudinally extensive transverse myelitis (LETM) in a seropositive HIV patient. Clin Neurol Neurosurg. 2013;115:1873–1875. http://dx.doi.org/10.1016/j.clineuro.2013.03.007 23619533 10.1016/j.clineuro.2013.03.007

[15] Feyissa AM, Singh P, Smith RG Neuromyelitis optica in patients with co-existing human immunodeficiency virus infection. Mult SclerJ. 2013;19(10):1363–1366. http://dx.doi.org/10.1177/1352458513483891 10.1177/1352458513483891 23549434

[16] Sweeney BJ, Manji H, Gilson RJ, Harrison MJ Optic neuritis and HIV-1 infection. J Neurol Neurosurg Psychiatry. 1993;56:705–707. http://dx.doi.org/10.1136/jnnp.56.6.705 8509790 10.1136/jnnp.56.6.705 PMC489626

[17] Babu K, Murthy K, Rajagopalan N, Satish B Vision recovery in human immunodeficiency virus-infected patients with optic neuropathy treated with highly active antiretroviral therapy: A case series. Indian J Ophthalmol. 2009;57:515–518. http://dx.doi.org/10.4103/0301-4738.53062 10.4103/0301-4738.53062 PMC2712706 19574705

[18] Burton BJL, Leff AP, Plant GT Steroid-responsive HIV optic neuropathy. J Neuroophthalmol. 1998;18:25–29. http://dx.doi.org/10.1097/00041327-199803000-00006 9532535

[19] Moodley A, Bhola S, Omar F, Mogambery J Antiretroviral therapy-induced Leber’s hereditary optic neuropathy. S Afr J HIV Med. 2014;5(2):69–71. http://dx.doi.org/10.7196/sajhivmed.1056

[20] Bhigjee AI, Madura S, Bill PLA, et al Spectrum of myelopathies in HIV seropositive South African patients. Neurology. 2001;57(2):348–351. http://dx.doi.org/10.1212/WNL.57.2.348 11468329 10.1212/wnl.57.2.348

[21] Paul F, Jarius S, Aktas O, et al Antibody to aquaporin 4 in the diagnosis of neuromyelitis optica. PLoS Med. 2007;4:e133 http://dx.doi.org/10.1371/journal.pmed.0040133 17439296 10.1371/journal.pmed.0040133 PMC1852124

[22] Kang ES, Min J-H, Lee KH, Kim BJ Clinical usefulness of cell-based indirect immunofluorescence assay for the detection of aquaporin-4 antibodies in neuromyelitis optica spectrum disorder. Ann Lab Med. 2012;32:331–338. http://dx.doi.org/10.3343/alm.2012.32.5.331 22950068 10.3343/alm.2012.32.5.331 PMC3427820

[23] Ghezzi A, Bergamaschi R, Martinelli V, et al Clinical characteristics, course and prognosis of relapsing Devic’s Neuromyelitis Optica. J Neurol. 2004;251:47–52. http://dx.doi.org/10.1007/s00415-004-0271-0 14999489 10.1007/s00415-004-0271-0

[24] Koga M, Takahashi T, Kawai M, et al A serological analysis of viral and bacterial infections associated with neuromyelitis optica. J Neurol Sci. 2011;300:19–22. http://dx.doi.org/10.1016/j.jns.2010.10.013 21056429 10.1016/j.jns.2010.10.013

[25] Wingerchuk DM, Banwell B, Bennett JL, et al International consensus diagnostic criteria for neuromyelitis spectrum disorders. Neurology. 2015;85:1–13. http://dx.doi.org/10.1212/WNL.0000000000001729 10.1212/WNL.0000000000001729 PMC4515040 26092914

[26] Pröbstel A-K, Rudolf G, Dornmair K, et al Anti-MOG antibodies are present in a subgroup of patients with a neuromyelitis optica phenotype. J Neuroinflammation. 2015;12:46 http://dx.doi.org/10.1186/s12974-015-0256-1 25889963 10.1186/s12974-015-0256-1 PMC4359547

[27] Olindo S, Bonnan M, Merle H, Signate A, Smadja D, Cabre P Neuromyelitis optica associated with subacute human T-lymphotropic virus type 1 infection. J Clin Neurosci. 2010;17:1449–1451. http://dx.doi.org/10.1016/j.jocn.2009.12.024 20638847 10.1016/j.jocn.2009.12.024

[28] Pittock SJ, Weinshenker BG, Wingerchuk D, et al Autoimmune neurological accompaniments of neuromyelitis optica (NMO). Ann Neurol. 2006;60:S41.

[29] Pittock SJ, Lennon VA Aquaporin-4 autoantibodies in a paraneoplastic context. Arch Neurol. 2008;65(5):629–632. http://dx.doi.org/10.1001/archneur.65.5.629 18474738 10.1001/archneur.65.5.629

